# Advances in biosynthesis and metabolic engineering strategies of cordycepin

**DOI:** 10.3389/fmicb.2024.1386855

**Published:** 2024-06-05

**Authors:** Ting Peng, Jinlin Guo, Xinxin Tong

**Affiliations:** The Ministry of Education Key Laboratory of Standardization of Chinese Medicine, Key Laboratory of Systematic Research of Distinctive Chinese Medicine Resources in Southwest China, Resources Breeding Base of Co-Founded, College of Pharmacy, Chengdu University of Traditional Chinese Medicine, Chengdu, Sichuan, China

**Keywords:** *Cordyceps militaris*, cordycepin, microbial fermentation, biosynthesis, metabolic engineering

## Abstract

*Cordyceps militaris*, also called as bei-chong-cao, is an insect-pathogenic fungus from the Ascomycota phylum and the Clavicipitaceae family. It is a valuable filamentous fungus with medicinal and edible properties that has been utilized in traditional Chinese medicine (TCM) and as a nutritious food. Cordycepin is the bioactive compound firstly isolated from *C. militaris* and has a variety of nutraceutical and health-promoting properties, making it widely employed in nutraceutical and pharmaceutical fields. Due to the low composition and paucity of wild resources, its availability from natural sources is limited. With the elucidation of the cordycepin biosynthetic pathway and the advent of synthetic biology, a green cordycepin biosynthesis in *Saccharomyces cerevisiae* and *Metarhizium robertsii* has been developed, indicating a potential sustainable production method of cordycepin. Given that, this review primarily focused on the metabolic engineering and heterologous biosynthesis strategies of cordycepin.

## 1 Introduction

*Cordyceps militaris*, belonging to the genus *Cordyceps* of Ascomycota phylum and the Clavicipitaceae family, is among the most important “traditional medicines” with a long history of medicinal use in China. This highly valued medicinal fungus has various clinical components, including cordycepin, carotenoid, D-mannitol, polysaccharides, ergosterol and mannitol, which have a wide range of pharmacological benefits, such as anticancer, antioxidant, antibacterial, and immunomodulatory effects. Among them, cordycepin, or 3′-deoxyadenosine, is a derivative of the nucleoside adenosine. Cordycepin was initially isolated from *C. militaris* by Cunningham et al. (1950) and is the main bioactivity ingredient in this fungus. In reality, except for *C. militaris*, cordycepin can also be produced by other cordyceps genus, such as *Cordyceps kyushensis* Kob and *Cordyceps cicadae*.

Cordycepin is recognized as a bioactive metabolite with therapeutic potentials, exhibiting an extensive range of biology activities, such as anti-cancer, anti-diabetic, anti-hyperlipidemia, anti-inflammatory, anti-photoaging, anti-microbial, anti- platelet aggregation, hypolipidemic, analgesic and immunomodulatory (Sharma et al., 2022). Recent study showed that cordycepin had a strong binding affinity with SARS-CoV-2 spike protein (interaction score: −145.3) and main proteases (M pro, interaction score: −180.5). It was also confirmed to have inhibitory potential against the COVID-19 polymerase enzyme (RdRp) (Verma, 2022), implying cordycepin has the potential for the treatment of COVID-19.

Nevertheless, due to its trace amount in natural sources, the production of cordycepin remains a challenge and is still far from the market demand. As a result, the global market prices of cordycepin gradually rose, approximately $440,000/kg in 2022 (Yang et al., 2020). In recent decades, considerable efforts were focused on the improvement of cordycepin productivity though chemical synthetic and biosynthesis methods. e.g., Masuda et al. (2014) have found that after 45 days (ds) of cultivation, the highest synthesis of cordycepin in the *C. militaris* mutant G81-3 can reach 14.3 g/L. The late-breaking study enclosed a single gene cluster containing four genes (*Cns1-4*) capable of synthesizing cordycepin and pentostatin (PTN). Cordycepin was mainly synthesized by the dephosphorylation of adenosine or its 2′, 3′ cyclic monophosphate (2′,3′-cAMP) to adenosine 3′- monophosphate (3′-AMP) catalyzed by Cns2, which was followed by oxidoreduction reactions by Cns1 (Xia et al., 2017). Based on the main biosynthetic pathways of cordycepin, synthetic biology approaches were gradually investigated to introduce these key enzyme genes into the expression system of various hosts to increase the yield and titer of cordycepin. The following review focused on the biosynthesis and metabolic engineering strategies, including microbial fermentation, mutation breeding, modification of pathways enzymes, genome-scale metabolic model (GEMs), etc.

## 2 Strain fermentation

### 2.1 Mutation breeding

**Figure 1:**
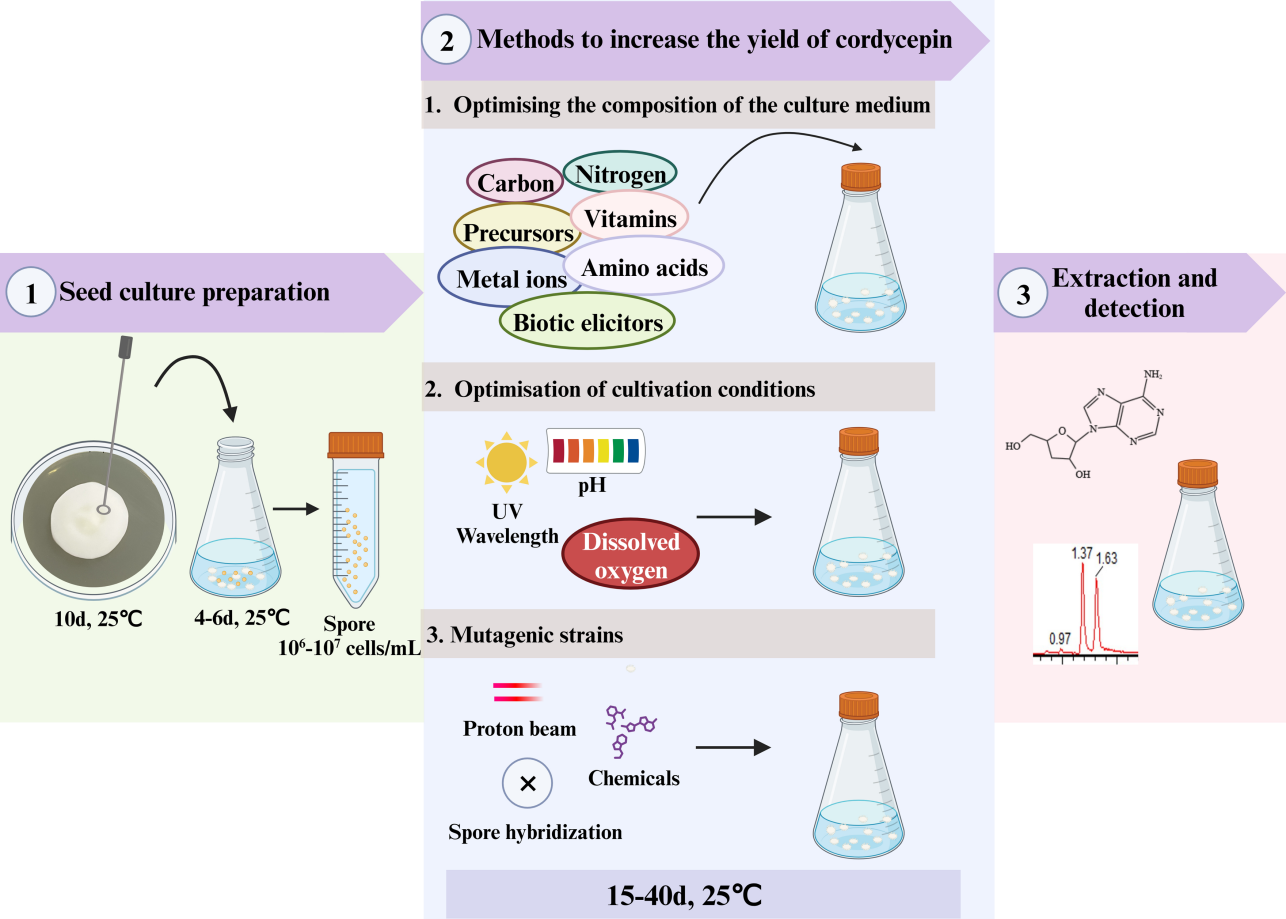
The methods for increasing *C. militaris’s* cordycepin output.

Cordycepin was mainly obtained from the fruiting bodies of *C. militaris*, with the limited concentration of 2–3 mg/kg (Yang et al., 2020). Relying solely on fermentation may not be sufficient to meet the significantly increased market demands for cordycepin. Mutation breeding is a technique that induces genetic mutations in organisms to create new traits. *C. militaris* mutants with increased cordycepin yield was obtained using mutation breading (Figure 1 and Table 1).

**Table 1:** Microbial fermentation and mutation breeding methods for enhancing cordycepin yield.

Fermentation condition’s category	Samples	Strains	Condition	Cordycepin production (mg/L, *(mg/g))	Increasement	Ref
Carbon Source	Fermented mycelium of *C. militaris*	–	40 g/L glucose	245.7	43.38% higher than 70 g/L glucose	Zhao et al. (2019)
		TBRC7358	Glucose	249.74	17.41% higher than sucrose	Lin et al. (2018)
Nitrogen source	Fermented mycelium of *C. militaris*	–	40 mM NH_4_^+^	420.5	70% higher than batch cultivation	Sung et al. (2010)
C/N ratio	Fermented mycelium of *C. militaris*	–	C/N = 1:1	345.4	40.5% higher than C/N = 5:1	Zhao et al. (2019)
Growth factors	*C. militaris* fruiting body	MS05	Addition of 4 g/L NAA	6.759 *	4.6 times	Raethong et al. (2018)
	Fermented mycelium of *C. militaris*	CGMCC2459	10 mg/L VB_1_	1159.341	95.8% higher	Mao et al. (2005)
Metal ions	Fermented mycelium of *C. militaris*	–	1 g/L FeSO_4_·7H_2_O	596.59	70% higher than no-add group	Shih et al. (2007)
		–	Addition of 10 mM Zn^2+^	1553.21	6.88 times more than the Zn-free sample	Wang K. et al. (2022) and Wang L. et al. (2022)
Culture conditions	Fermented mycelium of *C. militaris*	BCRC34380	Blue light (440–450 nm)	3,480	58% higher	Raethong et al. (2020)
		CCRC 32219	pH 4.0	315.2	2.28 times more than the pH 7.0	Zhao et al. (2020)
		-	4d: 50–60% DO, 9d: 30% DO	201.1	15% higher than control	Wongsa et al. (2020)
Mutagenic strains	Fermented mycelium of *C. militaris*	GYS60	Multifunctional plasma mutation system	7,883	20 times	Mao and Zhong (2004)
		NBRC 9787	Proton beam irradiation	7,400	64.86% higher	Masuda et al. (2014)
		CGMCC3.3149	Ultrasonic mutagenesis in conjunction with diethyl sulfate	1760	40% higher	Kang et al. (2014)
	Fermented mycelium of *C. kyushuensis*	*–*	Genome shuffling by UV and HNO_2_ mutagenesis	0.9783*	9.624 times	Wang et al. (2023)
	*C. militaris* fruiting body	Cm-B8	UV mutagenesis to treat the protoplasts	4.51*	1.74 times	Gu et al. (2007)
	Fermented mycelium of *C. militaris*	–	Spore hybridization	6.63*	116.67%	Fan et al. (2012)

NAA: 1-Naphthaleneacetic acid,VB1: Vitamin B1, DO: Dissolved oxygen: NA.

Through a multifunctional plasma mutation system (MPMS), the high cordycepin-yielding mutant strain GYS60 yielded about 7.883 mg/mL cordycepin, over 20 times higher than that in the wild strain *C. militaris* G81-3 (Zhang et al., 2020). By exposing to a proton beam irradiation, 14.3 g/L of cordycepin was yielded without crystallization via the former method (Masuda et al., 2014). Wang L. et al. (2017) and Wang Y. et al. (2017) obtained xanthine-guanine double auxotrophic mutants through ultrasonic mutagenesis in conjunction with diethyl sulfate treatment, improving the yield of cordycepin by 40% to 1.76 g/L on 20 ds after batch fermentation. The potential causes may be the obstruction of xanthine and guanine synthesis and metabolism, which resulted in an augmented metabolic flux directed towards the precursor substances adenine or adenosine (Wang L. et al., 2017; Wang Y. et al., 2017). Genome shuffling is a powerful technology that can rapidly improve the microbial phenotypes without revealing the genetic background. This method has been successfully used to improve tyrosine production in *Streptomyces fradiae*. Six cordycepin-improved *C. militaris* strains were screened using UV and HNO_2_ mutagenesis. The maximum cordycepin output was 978.25 μg/g, 9.62-fold increase over the ancestor strain (Wang L. et al., 2017; Wang Y. et al., 2017). Besides, a great number of strains with a high yield of target metabolites were created through rounds of protoplast fusion. For example, the cordycepin content of *C. militaris* mycelium generated using protoplast fusion technique was enhanced to 1.73 times of that in the parent strains (Zhou and Bian, 2007). Using UV mutagenesis on *C. militaris* protoplasts, cordycepin content increased to 4.51 mg/g, 1.74 times that of the parent strain (Liu T. et al., 2018; Liu H. et al., 2018). Regardless of the controversial issues surrounding genetically modified organisms or food, mating of parental strains remains a highly effective approach for enhancing cordycepin production. Kang et al. (2017) used spores hybridization to obtain the *C. militaris* KSP8 with a high cordycepin yield of about 6.63 mg/g and an increase of nearly 35% compared to parent strains.

Overall, mutation breeding has widely used in conjunction with other techniques such as genetic engineering and optimal fermentation conditions to improve the cordycepin yield and fulfill rising market demand for this valuable compound.

### 2.2 Fermentation conditions optimization

Cordycepin is naturally produced in *C. militaris*, *Aspergillus nidulans*, *C. kyushuensis* and *C. cicadae* (Kaczka et al., 1964; Liu T. et al., 2018; Liu H. et al., 2018; Zhao et al., 2019; Wang K. et al., 2022; Wang L. et al., 2022). In most previous studies, cordycepin was enhanced by solid state fermentation, submerged fermentation, and surface/static fermentation. Changes in the culture medium composition have obviously effect on the growth and quality of *Cordyceps* (Sung et al., 2010).Rice media, produced the maximum cordycepin (814.60 mg/g) followed by oat and wheat media (638.85 and 565.20 mg/g, respectively) (Adnan et al., 2017). To better understand the causes, the effect of fermented factors on cordycepin synthesis, including nutrients in the basic medium, physics conditions (light, temperature) and various exogenous substances (nitrogen sources, carbon sources, precursor of cordycepin synthesis, plant growth hormone, salt, vegetable oil) (Zhao et al., 2020), were systematically investigated and optimized (Figure 1 and Table 1).

Carbon sources are directly associated with the biosynthesis of several metabolites in fungal cells (Ruiz et al., 2010). Hexose, a six-carbon (C6) sugar, such as glucose, has been shown to preferred carbon source for cordycepin production due to the relationship between high sugar intake and cordycepin accumulation. The maximum cordycepin production (about 245.7 mg/L) was produced in the media containing 40 g glucose/L after 18 ds of culture (Mao et al., 2005). Another study found that the highest titer of cordycepin was produced in the medium broth with xylose compared to sucrose and glucose. Whereas, prolonged cultivation was required to achieve the extracellular cordycepin titer in xylose. Transcriptomic analysis revealed that the assimilated xylose might be directed towards cordycepin biosynthetic pathway rather than central carbon metabolism (Raethong et al., 2018).

Apart from the carbon source, nitrogen supply also aids in increasing cordycepin synthesis. Through the optimization of the feeding time and amount of NH_4_^+^, cordycepin concentration achieved to 420.5 ± 15.1 mg/L, and 70% higher compared to batch cultivation methods (Mao and Zhong, 2006). Besides, animal-free nitrogen source (vegetable seed extract), was also investigated. The addition of 80 g/L soybean extract powder (SBEP) increased the cordycepin production up to 2.52 g/L, which was greater than the control (peptone). Quantitative polymerase chain reaction (qPCR) result revealed that 80 g/L SBEP significantly increased the expression of genes linked with the carbon metabolic pathway, amino acid metabolism, and two crucial genes involved in the cordycepin biosynthesis (*Cns1* and *NT5E*) compared to peptone-supplemented culture (Sripilai et al., 2023).

Balance carbon and nitrogen metabolism is essential for metabolic system. The ratio of carbon and nitrogen in the media are also considered to be crucial for the cordycepin production. For examples, the maximal cordycepin production obtained was 345.4 mg/L with 42.0 g/L glucose and 15.8 g/L peptone (Mao et al., 2005). Adenosine and cordycepin content rose with a C/N ratio of 1:1.5, decreased with a C/N ratio of 1:3 (Shih et al., 2007). Cordycepin production increased to 2.64 g/L at a working volume of 147.5 mL, an inoculum size of 8.8% v/v, and a cultivation time of 40.0 ds with an 8:1 C/N ratio and 80 g/L SBEP supplementation (Raethong et al., 2020).

Biotic elicitors are crude extracts and purified products derived from fungal, plant and other sources (Halder et al., 2019). Adding biotic elicitors is an effective strategy to enhance cordycepin accumulation in some fungi. Naphthalene acetic acid (NAA) and vitamin are plant growth regulators with physiological effects with similar to plant hormone auxin (IAA) in terms of regulating plant growth and development. The addition of NAA to the solid medium of *C. militaris* significantly enhanced cordycepin content in the fruit-body. The addition of 10 mg/L Vitamin B_1_ (VB_1_) resulted in the highest yield of cordycepin, about 1.16 g/L after 35 ds of culture, approximately 95.8% higher than that control group (Kang et al., 2014). Through genes expressions and metabolome analysis, cordycepin synthesis might be associated with the metabolic pathways, including purine metabolism, TCA cycle, pentose phosphate pathway, alanine, aspartate, and glutamate metabolism, histidine metabolism and ABC transporter pathway (Wang et al., 2023). Besides, 5 biotic elicitors in different concentrations (0.05, 0.5, 1, and 2 mg/mL), including chitosan (CHT), 2,4-dichlorophenoxyacetic acid (2,4-D), methyl jasmonate (MeJA), gibberellic acid (GA), and triacontanol (TRIA), were firstly introduced to enhance the production of 10 major components in the fruit-body of *C. militaris,* showing that the addition of 1 mg/L of chitosan resulted in a 0.34-fold increase in cordycepin production compared to control (Lin et al., 2020). It was demonstrated that the application of biotic elicitors is an effective strategy to enhance the production of bioactivity components and the effect of biotic elicitors depended on their types and concentrations.

Trace metals have been reported to have a significant effect on the growth of *C. militaris*. For instances, 0.1 mM Mn^2+^ stimulated cell growth and promote the synthesis of Ns, such as uridine, guanosine, and adenosine (Gu et al., 2007). The yield of cordycepin reached to 596.59 mg/L after initially adding 1 g/L FeSO_4_·7H_2_O, roughly 70% higher than control, which might be related to the rapidly decreased consumption of inosine 5′-monophosphate (IMP), a putative cordycepin precursor (Fan et al., 2012). Besides, the addition of zinc (Zn) resulted in cordycepin reaching 1.55.g/L after 15 ds of cultivation at 25°C, 6.88 times higher than the control (225.66 mg/L). It suggested that Zn was involved in global transcriptional control and influenced cordycepin biosynthesis (Zhao et al., 2020). These findings underlined the importance of trace metals in modulating cellular processes and enzyme activities in *C. militaris*, enhancing their roles in the fungus’s growth and metabolic functions.

In engineering process, physical parameters such as pH, dissolved oxygen, light, temperature and biotic elicitors were extensively exploited to improve cordycepin yield. Adnan et al. (2017) found that the optimal conditions for maximum cordycepin production were 25°C, pH5.5 and 21 ds of culture. *C. militaris* liquid culture was exposed to light-emitting diode (LED) blue light (440-450 nm) for 16 h per day, significantly increasing cordycepin accumulation (Lin et al., 2018).

## 3 Biosynthesis pathways of cordycepin

Cordycepin has a complex metabolic pathway and synthetic regulation that has yet to be fully disclosed. Kredich and Guarino (1961) firstly proposed adenosine as a potential precursor of cordycepin biosynthesis in *C. militaris*. Lennon and Suhadolnik (1976) hypothesized that the mechanism for cordycepin synthesis might be similar to that of 2′-dA. The biosynthesis of cordycepin was investigated using [U-^14^C] adenosine and [3-^3^H] ribose. It was proposed that cordycepin can be produced by converting adenosine to 3′-deoxyadenosin (dA) without hydrolyzing the N-riboside bond, similar to 2′-dA synthesis (Yang et al., 2020). Lin et al. (2016) observed a significant up-regulation of 5′-nucleotidase (5’-NT) expression in *Ophiocordyceps sinensis* through qPCR analysis, indicating the potential regulatory role for 5’-NT in cordycepin production.

Based on transcriptomics analysis, Xiang proposed that adenosine kinase (ADK), adenylate kinase (ADEK) and 5′-NT, which are involved in phosphorylation and dephosphorylation in the adenosine metabolism pathway, may also be involved in phosphorylation and dephosphorylation in the cordycepin biosynthesis pathway and additional subunits also be involved in the reduction mechanism of the cordycepin synthesis (Xiang et al., 2014). Ribonucleotide reductases small subunit (RNRM) expression significantly increased in *C. militaris* with the high yield of cordycepin compared to that in wild (Liu G. et al., 2020; Liu W. Y. et al., 2020). The proposed pathway of cordycepin production in *C. militaris* is described as: Adenylate kinase (AK) converts AMP to adenosine diphosphate (ADP), which is then catalyzed by ribonucleotide reductase (nrdJ) to 3′-deoxyadenosine 5′-diphosphate (3’-dADP). Next, AK converts 3’-dADP to 3′-deoxyadenosine 5′-phosphate (3’-dAMP), which is further converted to cordycepin by 5′-nucleotidase. Additionally, the involvement of 3′, 5′-cyclic-nucleotide phosphodiesterase (PDEs) was proposed to be involved in the synthesis of cordycepin in *Paecilomyces hepialid* through the differential gene expression analysis (Pang et al., 2018). The addition of 5 mg/L rotenone significantly increased cordycepin production by 316.09%, along with mycelial growth inhibition and cell wall destruction. The integrated analysis of the two omics confirmed that rotenone altered the nucleosides metabolism pathway toward adenosine and activated the genes responsible for producing cordycepin (*cns1-3*) from adenosine (Ma et al., 2023). The L-alanine addition to *C. militaris* fermentation increased cordycepin yield by up-regulating the transcriptome level of genes involved in the bio-pathways of energy generation and amino acid conversion. And two Zn2Cys6-type transcription factors, CmTf1 and CmTf2, were also found to be highly up-regulated after the addition of L-alanine, probably participating into cordycepin metabolism (Chen et al., 2020).

Metabolomics studies showed that many metabolic pathways have been changed in the high-yielding cordycepin strains, e.g., citric acid cycle, purine metabolism, and pyrimidine metabolism (Ma et al., 2023). Changes in polysaccharide and protein synthesis metabolic poly-pathways, such as galactose metabolism and amino acid metabolism, would boost cordycepin′s production (Yu et al., 2023). Besides, the global metabolic response of *C. militaris* strain TBRC6039 in cordycepin synthesis on diverse carbon sources, namely sucrose, glucose and xylose, were investigated using transcriptome analysis and the genome-scale network-driven analysis. A gene (CL1730.Contig2) encoding ATP phosphoribosyltransferase (ATPPRT) was found to be highly expressed in xylose containing conditions, suggesting that ATPPRT might function in the biosynthesis of cordycepin (Raethong et al., 2018). Xia et al. (2017) also found that ATPPRT might be associated with PTN biosynthesis when xylose and phosphoribosyl pyrophosphate (PRPP) and adenosine were used as substrates.

**Figure 2:**
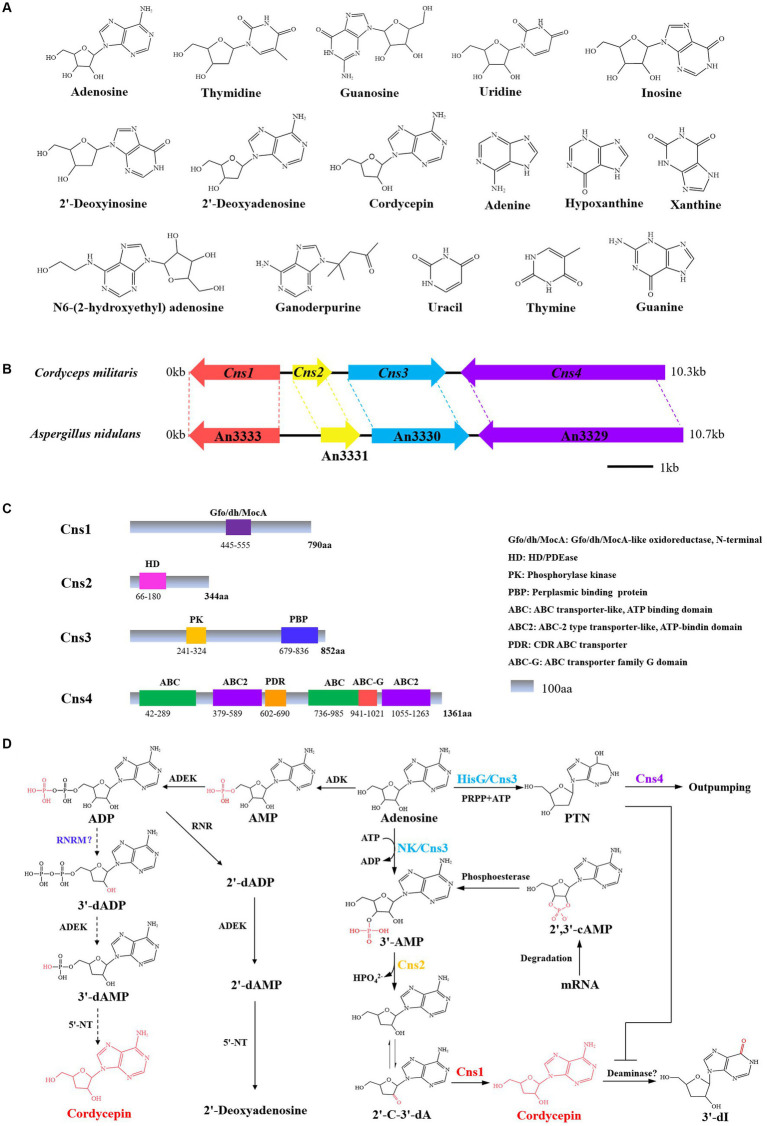
Cordycepin biosynthetic gene cluster and biosynthesis pathway. **(A)** Chemical structure formula of Nt/Ns. **(B)** The gene cluster for synthesize cordycepin in *C. militaris* and *A. nidulans*. **(C)** Schematic structure of the proteins Cns-4. **(D)** Biosynthetic pathway of cordycepin. PRPP, phosphoribosyl pyrophosphate; 2′-C-3′-dA, 2′-carbonyl-3′-deoxyadenosine; NK, an N-terminal nucleoside kinase; HisG, a C-terminal HisG family of ATP phosphoribosyltransferases; 3′-dI, 3′-deoxyinosine.

Xia et al. (2017) found that *C. militaris* possesses a newly evolved gene cluster of Cns1-Cns4, which mediate the dual biosynthesis of cordycepin and another adenosine analog, PTN, gene locations and protein structural domains are shown in Figure 2. Cordycepin biosynthesis starts from adenosine and proceeds through the stepwise reactions of phosphorylation, dephosphorylation, and reduction catalyzed by Cns1/Cns2 complex. Cns1 and Cns2 interact tightly with each other and that one enzyme cannot function alone. Cns1 and Cns2 are required for cordycepin biosynthesis, while the Cns3 gene is involved in biosynthesis of PTN. The Cns1/Cns2 complex catalyzes cordycepin biosynthesis in parallel with PTN biosynthesis. This dual production is analogous to the bacterial “protector-protégé strategy” of purine metabolism, in which the protected cordycepin can be deaminated to 3′-deoxyinosine once it reaches a self-toxic level in fungal cells. Cns4 regulates the cell’s outpumping of PTN. So, the biosynthesis of cordycepin is coupled with PTN production by a gene cluster and the coupling is important for metabolic regulation where PTN safeguards cordycepin from deamination by inhibiting adenosine deaminase (ADA) activity (Xia et al., 2017). The requirement of multienzyme complexes for purine biosynthesis has long been known. Based on the proposed biosynthesis principles, *C. militaris* was engineered to produce higher levels of cordycepin and PTN. Besides, ADA is widely distributed in mammalian blood and tissues, which caused that cordycepin is quickly metabolized and converted into the inactive metabolite upon entering the body. Guan constructed a non-competitive inhibition to screen ADA inhibitors against the degradation of cordycepin via UHPLC–MS/MS. Oleanolic acid and ursolic acid from *Ligustri Lucidi* Fructus were chosen as ADA inhibitors with half maximal inhibitory concentration of 21.82 ± 0.39 and 18.41 ± 0.14 μM, respectively (Guan et al., 2020). Another research discovered that emodin inhibits ADA, as well as a high cytotoxic activity against K562 leukemia cells (Zhang et al., 2019). The proposed biosynthetic pathway of cordycepin in *C. militaris* is described as:using adenosine as the starting material, the nucleoside / nucleotide kinase domain of Cns 3 catalyses the hydroxyl phosphorylation of adenosine at 3’-OH site to yield adenosine-3′-monophosphate (3’-AMP). Meanwhile, 3’-AMP could also be produced from 2′, 3′-cyclic monophosphate (2′,3’-cAMP) by an unidentified enzyme. Cns2 then phosphohydrolates 3′-AMP to 2′-carbonyl-3′-deoxyadenosine (2’-C-3′-dA). 2’-C-3′-dA is then converted to cordycepin by oxidoreduction reaction via Cns1.

In addition to *C. militaris*, the orthologous genes also present in the genome of *A. nidulans* and *C. kyusyuensis*. It was suggested that the both species can synthesize cordycepin. Through the high-throughput analysis of the transcriptome and proteome of *C. kyushuensis* Kobayasi, the gene cluster *ck1-ck4* was found to enhance the production of cordycepin and the biosynthesis pathways of cordycepin-PTN coupling was also confirmed (Zhao et al., 2019). United States FDA approved clinical trials of cordycepin combined with PTN for the treatment of terminal deoxynucleotide-positive leukemia (Foss, 2000). Furthermore, the modification of epigenetic status by valproic acid (VPA) significantly enhanced cordycepin production by altering key gene regulatory in *C. militaris*. The production of cordycepin peaked at 2,835.32 ± 34.35 mg/L in 400 mL working volume and increased by 41.187% after 50 μM VPA treatment, compared to the basal media. The explanation could be related to up-regulation of 5’-NT, ADEK, phosphoribosyltransferase (PRTase) and Cns1-Cns4 in *C. militaris* (Kunhorm et al., 2022). The proposed cordycepin biosynthetic pathways were sketched in Figure 2.

## 4 Cordycepin biosynthesis strategies

### 4.1 Genetic transformation and genome editing method

Traditional mutation breeding is untargeted, laborious and time-consuming. Based on the elucidation of cordycepin biosynthesis pathway, genetic engineering of the *Cordyceps* strains has a great potential in cordycepin synthesis. Genome/gene editing technologies methods were based on homologous recombination (HR), split-marker approach, *Agrobactirium tumfacience* mediated-transformant (ATMT)/protoplast-polyethylene glycol (PEG)-mediated transformation (PMT) random/targeted gene mutagenesis and Clustered Regularly Interspaced Short Palindromic Repeats (CRISPR) (Chen et al., 2018; Lou et al., 2018; Liu et al., 2023). The commonly used transformation methods are summarized in Table 2.

**Table 2:** Transformation protocols.

Transformation method	Gene editing technology	Recipient	Agrobacterium strain	Protoplasts preparation	Optimal transformation conditions	Tranformation Efficiency	Ref
ATMT	Random insertion	*O. sinensis* KD1223, blastospores	LBA440, AGL-1, GV3101	–	100 μL induced *A. tumefaciens* (OD_600_ 0.6–0.8) mixed with 1 × 10^6^ /100 μL blastospores, 200 μM AS at 16°C in the dark for 96 h, selected on PPDA media 300 μg/mL Cef,100 μg/mL Amp and 300 μg/mL Hyg at 13°C for 60 ds.	43.75%	Liu G. et al. (2020) and Liu W. Y. et al. (2020)
	Random insertion	*C. militaris* conidia	AGL-1	–	100 μL induced *A. tumefaciens* (OD_600_ 0.6–0.8) mixed with 1 × 10^4^/100 μL conidia, 200 μM AS at 23°C in the dark for 48 h, selected on PPDA media with 400 μg/mL, Hyg 200 μg/mL Cef and 60 μg/mL Str sulphate at 23°C for 5 ds.	30–600 transformants per 1 × 10^5^ conidia	Zheng et al. (2011)
	HR-based targeted mutagenesis	*C. militaris* 40 conidia	AGL-1	–	100 μL induced *A. tumefaciens* (OD_600_ 0.6–0.8) mixed with 1 × 10^4^ μL conidia, 200 μM AS at 23°C in the dark for 4–5 ds, selected on PPDA media with 300 μg/mL Cef and 500 μg/mL Hyg at 23°C for 10 ds.	-	Yang et al. (2016)
PMT	Random insertion	*C. militaris* CM10 mycelium protoplasts	–	4-day-old mycelia were incubated in an enzymolysis solution (pH 6.5, 1.00% lysing enzyme, 1.00% lywallzyme, and 0.8 M KCl at 32°C for 3.0 h	10 μg linear DNA mixed with 1 × 10^7^/100 μL protoplast on ice for 5 min, added with 50 mL of 25% PEG 4000 buffer on ice for 30 min, following 0.5 mL PEG 4000 buffer at 28°C for 20 min, combined with 1.0 mL STC buffer (18.2% sorbitol, 10.0 mM Tris–HCl) (pH 7.5), 25.0 mM CaCl2, w/v), finally, cultured on RM plates with 1.0 M sorbitol and 300 μg/mL Glu ammonium at 25°C for 10 ds.	87 positive mutants	Lou et al. (2019)
	Split‑Marker	*C. militaris* CM10 blastospores protoplasts		rinsed with 0.8 M KCland then digested with lywallzyme solution (25 mg/mL in 0.8 M KCl) at 24°C for 2.5 h	10 μg split-marker fragments mixed with 1 × 10^7^/100 μL protoplasts on ice for 5 min, added with 50 μL of 25% PEG 4000 buffer on ice for 30 min, next 0.5 mL PEG 4000 buffer at 28°C for 20 min, and then combined with 1.0 mL STC buffer, spread PPDA media containing 0.8 M mannitol and 300 μg/mL Glu ammonium at 25°C in the dark for 7–14 ds.	13.24%	Lou et al. (2018)
	CRISPR/Cas9 and HR-based mutagenesis	*C. militaris* CGMCC 3.16323, blastospores protoplasts		rinsed with 0.8 M KCl, and then digested with lywallzyme solution (25 mg/mL in 0.8 M KCl) with at 24°C for 2.5 h	1 × 10^7^/100 μL protoplast mixed with 2 μg plasmid incubation on ice for 5 min, added with 50 μL PEG buffer on ice for 30 min, next 0.5 mL PEG buffer at 28°C for 20 min, and then combined with 1 mL STC buffer, centrifuged at 3000 rpm for 10 min, resuspended in 200–400 μL STC buffer, spread on PPDA media with 1 M mannitol and Hyg at 25°C for 4–7 ds.	88.89%	Meng et al. (2022)

PMT, PEG-mediated transformation; HR, Homologous recombination; Cef, cefotaxime; Amp, ampicillin; Hyg, hygromycin; Str sulphate, streptomycin sulphate; Glu ammonium, glufosinate ammonium, “–“, NA.

ATMT has been widely and effectively used for transformation, random or targeted gene insertion in several filamentous fungi (Zheng et al., 2011). Compared to other transformation techniques, ATMT allows the flexibility with respect to choose of protoplasts, mycelium, conidia and other cell types as recipient for transformation (Sun et al., 2018). To obtain insertional mutagenesis in *C. militaris*, the transformants was successfully obtained by co-cultivation of *A. tumefaciens* AGL-1 carrying vector pATMT1 with the conidia of JM strain, with efficiency in the range of 30–600 transformants/1 × 10^5^ conidia and 55–80% of transformants harbored a single-copy (Zheng et al., 2011). The transformation efficiency can be optimized as ratio of bacteria to conidia, pH of co-culture media and co-culture duration.

PEG, an important method for studying gene function in filamentous fungi, has been successfully applied to *C. militaris* for transformation (Lou et al., 2019). The yield of protoplasts was significantly affected by the enzyme system used to digest the hyphae. In one study, the 4-day-old mononuclear *C. militaris* mycelia were incubated in an enzymolysis solution (pH 6.5) composing of 1.00% lyticase, 1.00% lywallzyme, and 0.8 M KCl at 32°C for 3.0 h, achieving the highest protoplast yield of 2.25 × 10^7^ protoplasts/g fresh weight. 10 μg of the linear DNA fragment and 100 μL of protoplasts were mixed, obtaining 87 PCR-positive mutants (Lou et al., 2019). Besides, the applications of remnerative fungi *A. cinnamomea* and *C. militaris* were expanded to promote health and well-being through the protoplasts fusion that can be developed as innovative materials for uses. The optimal digestion condition for the preparation of *C. militaris* protoplasts was 2.0% lywallzyme, 1.0% cellulase, 0.5 M KCl, temperature as 32°C and time as 2.5 h, achieving the highest yield of 1.10 × 10^8^ protoplasts/mL. Taken together, the primary factors influencing protoplast transformation include the enzyme digestion conditions (lytic enzymes, the concentration of the enzyme mixture, the concentration of KCl, digestion temperature, duration and pH), protoplasts regeneration, concentrations of PEG4000 and plasmid DNA.

However, random and HR-based targeted mutagenesis techniques were insufficient and too complicated to meet the requirement for precise, highly efficient and repeatable editing. The breakthrough discovery of Cns gene cluster integrated with targeted genome editing techniques, such as gene knockout and knockin. CRISPR technology is an innovative and highly efficient genome-editing tool capable of gene editing (Alamillo et al., 2023), presenting a promising approach to enhance cordycepin production and related compounds. CRISPR Type II is the most widely used, comprising of two key components: the CRISPR-Cas9 derived from *Streptococcus pyogenes* and a single-guide RNA (sgRNA) that combines precrRNA and tracrRNA (Mir et al., 2018). CRISPR-Cas9 system has been applied in filamentous fungi, such as *Aspergillus* (Nødvig et al., 2015; Vanegas et al., 2019), *Trichoderma reesei* (Liu et al., 2015), and *Neurospora crassa* (Matsu-ura et al., 2015), whereas its application in *Cordyceps* genus poses distinct challenges. *Cordyceps* genus undergo a complex two-stage growth process and face high rates of fruit-body gemmated degeneration. Above factors contribute to additional difficulties in implementing the CRISPR system in these species (Zhang et al., 2023). In *C. militaris*, the *pyrG* gene was successfully edited using CRISPR/Cas9 system, with the efficiency of 11.76%. The editing efficiency of *Cmura5* reached 100% when an optimized ribonucleoprotein (RNP)-based method was employed, indicating that CRISPR-Cas9-based gene editing techniques can be applied in this species. One study used a codon-optimized Cas9, along with a newly reported promoter (*Pcmlsm3*) and terminator (*Tcmura3*), to enhance Cas9 expression (Chen et al., 2018). Furthermore, a CRISPR/Cas9 system based on AMA1 sequence was also successfully constructed to edit multiple genes in *C. militaris,* with efficiencies of 55.1 and 89% for *Cmwc-1* and *Cmvvd*, respectively. Using this system, a single RNA was edited with an efficiency of 73.9%, while, double genes (*Cmwc-1* and *Cmvvd*) were edited simultaneously with an efficiency of 10% (Meng et al., 2022). CRISPR/Cas9 system can be applied to edit the biosynthetic pathway of cordycepin, the supply and use of adenosine, and PRPP utilization, increasing cordycepin production and facilitating the rapid development of the edible and medicinal fungi industry.

### 4.2 Cordycepin heterologous biosynthesis

Introducing microbes by virtue of synthetic biology holds a great potentially strategies for cordycepin synthesis. Cordycepin heterologous biosynthesis in *Saccharomyces cerevisiae*, *Yarrowia lipolytica*, and *Komagataella phaffii* uses an enzyme complex of Cns1 and Cns2 and 3′-AMP as the substrate. Cordycepin was enhanced through metabolic engineering strategies, i.e., promoter, protein, adenosine triphosphate, and precursor engineering (Song et al., 2023). *S. cerevisiae* S288c was successfully employed to synthesize cordycepin by introducing *ScCNS1* and *ScCNS2* cloned from *C. millitaris* L5111, and the titer reached 137. 27 mg/L after fermentation optimization (Huo et al., 2021). The emerging non-conventional yeasts, including *K. phaffii*, *Y. lipolytica*, *Kluyveromyces lactis*, *Kluyveromyces marxianus*, *Hansenula polymorpha* and *Rhodotorula toruloides*, have received a lot of attention these years and are being considered as alternatives for biorefinery platforms due to their inherent qualities, ranging from a broad spectrum of carbon-source usage to high environmental tolerance (Patra et al., 2021). *Y. lipolytica*, an FDA-approved non-conventional yeast, is regarded as a possible platform for manufacturing non-native compounds due to its unique properties (Wang K. et al., 2022; Wang L. et al., 2022). *Y. lipolytica* has gained particular interest as chassis strains in industry due to the ability of the biosynthesis of value-added molecules by utilizing low-cost bioresources. Moreover, the 3′-AMP biosynthetic pathway naturally present in *Y. lipolytica,* which is critical to the synthesis of cordycepin. One study showed that the engineered *Y. lipolytica* produce cordycepin as high as 3.26 g/L from glucose with the metabolic engineering strategies, including the optimizing enzyme fusion and the integration sites, the precursor supply, purine biosynthetic pathway optimization through modular engineering, and using glucose and molasses as the carbon source (Duan et al., 2022). This study shows that the exiting potential of non-conventional yeasts in producing cordycepin and other Ns antibotics using artificial microbial cell factories. The maximum cordycepin productivity of 656.27 mg/L/d (72 h) and cordycepin titer of about 2.29 g/L (120 h) was produced from agro-industrial waste by *Y. lipolytica* in the optimized media. This study lays the groundwork for effective cordycepin production from agro-industrial waste (Duan et al., 2023). In recent study, the production titer and yields of cordycepin in the engineered *Y. lipolytica* YlCor-18 under fed-batch fermentation were enhanced to 4.36 g/L and 213.85 mg/g after 168 h of culture (Song et al., 2023). Based on above studies, *Y. lipolytica* was considered to be a cell factory for cordycepin production and also serve as the model for the green biomanufacturing of other nucleoside antibiotics employing artificial cell factories. Besides, *K. phaffii* has the intriguing ability to efficiently digest methanol as a sole source of carbon and energy. It was engineered to biosynthesize cordycepin from methanol, which could be converted from CO_2_. By implementing the fermentation optimization, cordycepin content in broth reached 2.68 g/L within 168 h. This corresponded to a productivity rate of about 15.95 mg/L/h (Tan et al., 2023). The production of cordycepin in strains through heterologous biosynthesis is shown in Table 3. Besides, Xia et al. (2017) successfully expressed Cns2 in *E.coli*, but failed to express Cns1. *In vitro* attempts to validate the feasibility of Cns2 directly converting 3’-AMP to 2’-C-3′-dA were unsuccessful, validating that Cns2 functions in the presence of Cns1 (Xia et al., 2017). Various certain drawbacks, such as biased codon usage, protein solubility, mRNA stability, and secondary structure, may hinder the *E. coli* system from being employed efficiently to overexpress heterologous genes (Gu et al., 2010; Pellizza et al., 2018).To address the potential challenges, some engineering strategies will be adopted across several sizes, such as replacing rare codons with more favorable major codons and periplasmic secretion of heterologous proteins, which provides several benefits, including proper folding, solubility, ease of purification, and enhanced protein output (Terpe, 2006).

**Table 3:** The production of cordycepin in strains through heterologous biosynthesis.

Engineered Strains	Biosynthesis strategies	Optimal fermentation conditions	Cordycepin yield	Increasement	Ref.
*S. cerevisiae* S288c	heterologous expression of *ScCNS1* and *ScCNS2* cloned from *C. millitaris*	50 g/L initial glucose, and then supplemented with 5 mmol/L Cu^2+^ and 1.0 g/L adenine, 5 L stirred fermenter cultured for 144 h	0.14 g/L	240% higher than that of the non-optimized fermentation system	Huo et al. (2021)
*Y. Lipolytica* YL-C19	heterologous expression of Cns1 and Cns2 cloned from *C. militaris*, enzyme fusion and integration site engineering optimizing metabolic flow in the purine biosynthetic pathway, supplying adenosinea and using a combination of glucose and molasses as the carbon source	YY base medium (9 g/L yeast extract, 8 g/L yeast nitrogen base without amino acids), 20 g/L glucose, and 9% (v/v) AHM, pH 5.5, and cultivated for 144 h	3.58 g/L	4.3 times higher compared with non-optimized fermentation system	Duan et al. (2022)
*Y. lipolytica* YL-C14-4	heterologous expression of Cns1 and Cns2 cloned from *C. militaris*, modification of the glycolysis pathway and PPP pathway by co-overexpression of *GLK1, PGM1, PRS1*, and *PRS3*	25% (v/v) AHM, 18% (v/v) waste spent *Y. lipolytica*, 0.54 g/L DHP with C/N molar ratio of 120:1, initial pH of 5 and cultured for 72 h	2.29 g/L after 120 h, 656.27 mg/L/d after 72 h	28.81% higher compared with the original medium	Duan et al. (2023)
*lipolytica* YlCor-18	heterologous expression of *CmCns1* and *CmCns2* cloned from *C. militaris*, combined with metabolic engineering strategies including promoter, protein, adenosine triphosphate, and precursor engineering	30 g/L glucose, 1.0 g/L adenine in YNB medium, and cultivated for 168 h	4.36 g/L and 213.85 mg/g, respectively, after 168 h	2.1 times higher compared with non-optimized fermentation system	Song et al. (2023)
*K. phaffii*	heterologous expression of *CmCns1* and *CmCns2* cloned from *C. militaris*	0.5% (v/v) methanol, 2.0 g/L adenine, starting biomass of OD_600_ = 5.0, pH 4.0 or 5.0, and cultivated for 168 h	2.68 g/L within 168 h, abour 15.95 mg/(L.h)	2.7 times higher compared with non-optimized fermentation system	Tan et al. (2023)

ScCNS1, heterologous expression of CNS1 in *S. cerevisiae*; ScCNS2, heterologous expression of CNS2 in *S. cerevisiae*; PPP, pentose phosphate pathway; GLK1, glucokinase gene; PRS1/3 phosphoribosylpyrophosphate synthetase gene 1/3; PGM1, phosphoglucomutases gene; AHM, acid hydrolyzed molasses; DHP: diammonium hydrogen phosphate; YNB, yeast nitrogen base.

**Figure 3:**
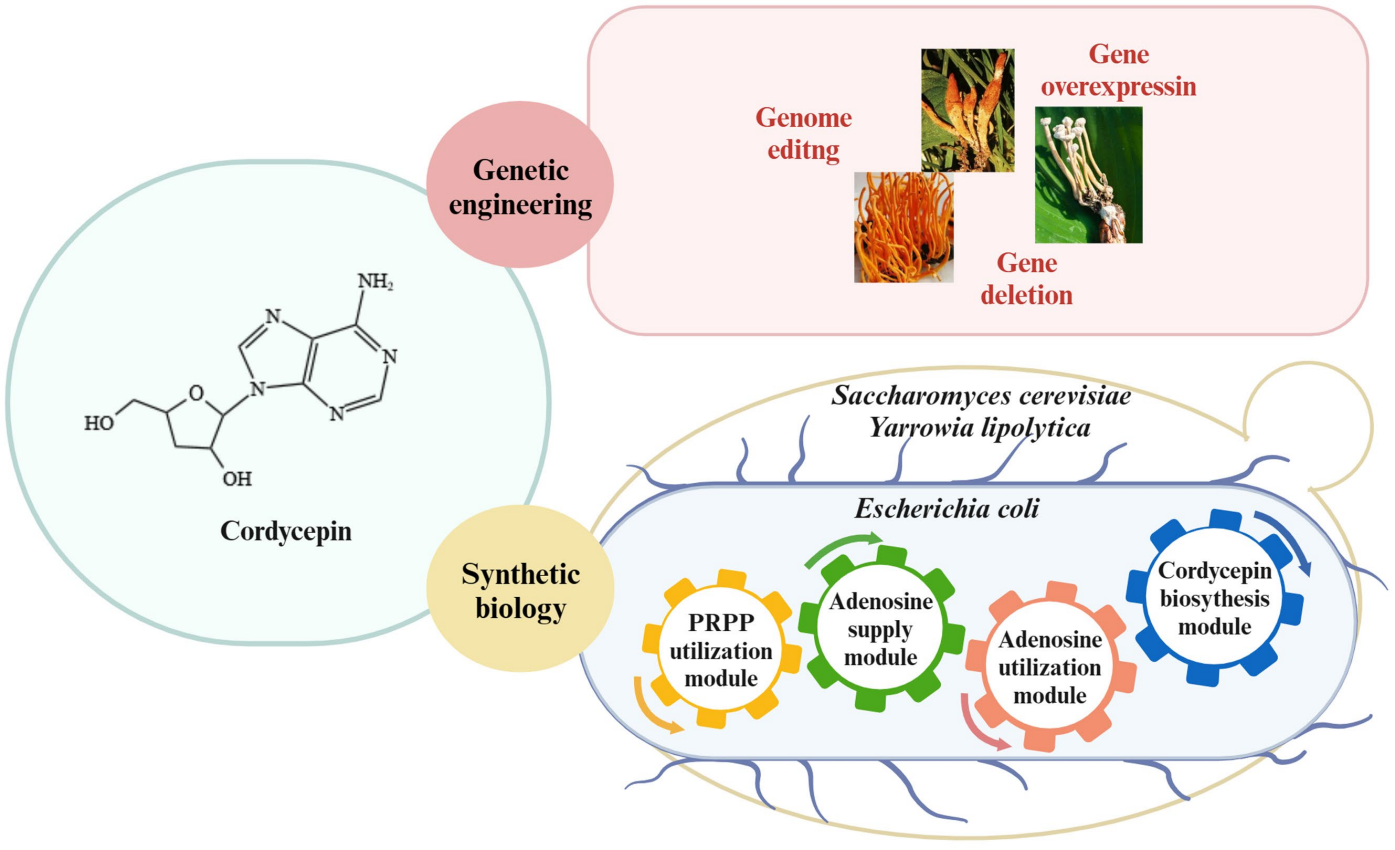
Strategies for cordycepin biosynthesis. Enhancement of cordycepin content in *Cordyceps* spp. through genetic engineering; synthesis of cordycepin in *E. coli*, *S. cerevisiae* and *Y. lipolytica* through synthetic biology.

Therefore, cordycepin yield will be enhanced through editing the pathways of cordycepin production in *C. militaris* and, on the other hand, cordycepin can be also heterologous biosynthesized by introducing the cordycepin biosynthetic pathway into engineered yeast and *E. coli* strains (Figure 3). Metabolic engineering strategies and the fermentation conditions will be combined to enhance the synthetic capability of engineered strains.

## 5 *In vitro* metabolic engineering (ivME)

ivME is the process of assembling many purified or partially purified enzymes along with nonenzyme components in accordance with the designed pathways to convert certain substrates into target compounds *in vitro* (Wei et al., 2020). ivME offers advantages such as precise reaction control and increased product yield, eliminating the need for complex cell metabolism engineering and route development. ivME has been used to investigate functional enzymes and metabolites, such as camalexin, mevalonate and isobutanol (Taniguchi et al., 2017). In 2019, a new *in vitro* approach for cordycepin synthesis was devised and announced as a patent for innovation [CN109628528A] by adding cordycepin synthesis-related enzymes from *C. militaris* and substrates. However, ivME encounters the problem of enzyme inactivation, the complexity of the experimental techniques, and adenosine substrate inefficiency. This system is challenging to scale up for biomanufacturing. So a combined *in vivo*/*in vitro* metabolic engineering strategy was proposed to accelerate the potential for increased cordycepin yield.

## 6 Conclusion and future prospect

The *Cordyceps* species, as the important sources of biochemical molecules and clinic drugs, has gained a considerable significance in several biotechnology, industrial and clinical applications. Among them, *C. militaris* was a promising candidate for the industrial applications of cordycepin production. Currently, most studies have focused on the fermentation strategies to increase cordycepin yield. Moreover, the integrated application of multi-omics, systems biology and synthetic biology techniques on *Cordyceps* allowed a much deeper understanding of the biology of *Cordyceps* and cordycepin synthesis, opening new and exciting approaches for exploring the biological and anabolic characteristics of cordycepin. Till now, some effective systems as useful tools of insertional mutagenesis in *C. militaris* have been successfully developed and utilized, including ATMT, PMT, particle bombardment (PB), split marker approach and CRISPR/Cas9. In particular, CRISPR/Cas9 has been successfully applied to precisely edit certain key genes in *C*. *militaris,* which will be used to edit the pathways of cordycepin production. It was reported that *Agaricus bisporus* with a CRISPR/Cas9-edited genome escaped the supervision of the US Department of Agriculture in 2016. CRISPR-based genome engineering technique led to a new era of the genome reconstruction of *C. militaris*, accelerating industrialization of mushroom.

Moreover, with the broad application of synthetic biology and the elucidation of cordycepin biosynthetic pathway, cordycepin can be heterologously biosynthesized in engineered yeast strains. Various system metabolic engineering methodologies, such as enzyme and precursor engineering, modular engineering and fermentation condition optimization, will be applied to enhance the synthetic potential of engineered strains. Besides, ivME is an alternative strategy that can overcome the limitations of the cell membrane and cell growth while displaying several advantages in terms of flexibility and control. ivME has been developed as an effective technique for synthesising functional enzymes and valuable chemicals, potentially leading to a high yield of cordycepin.

Therefore, the advancement of gene editing method, synthetic biology and *in vivo* / *in vitro* metabolic engineering would improve cordycepin synthesis and facilitate commercial-scale production.

## Author contributions

XT: Conceptualization, Writing – original draft, Writing – review & editing, funding acquisition, supervision. JG: Writing – review & editing, supervision. TP: Writing – original draft.

## Glossary

**Table:** 

ds	days
h	hour
PTN	pentostatin
2′,3′-cAMP	2′, 3′ cyclic monophosphate
3′-AMP	adenosine 3′- monophosphate
GEMs	genome-scale metabolic model
SBEP	soybean extract powder
qPCR	Quantitative polymerase chain reaction
NAA	Naphthalene acetic acid
VB1	Vitamin B1
CHT	chitosan
2,4-D	2,4-dichlorophenoxyacetic acid
MeJA	methyl jasmonate
GA	gibberellic acid
TRIA	triacontanol
IMP	inosine 5′-monophosphate
LED	light-emitting diode
MPMS	multifunctional plasma mutation system
dA	deoxyadenosin
5’-NT	5′-nucleotidase
ADK	adenosine kinase
ADEK	adenylate kinase
RNRM	ribonucleotide reductases small subunit
PDEs	phosphodiesterase
ATPPRT	ATP phosphoribosyltransferase
PRPP	phosphoribosyl pyrophosphate
ADA	adenosine deaminase
VPA	valproic acid
PRTase	phosphoribosyltransferase
HR	homologous recombination
ATMT	Agrobactirium tumfacience mediated-transformant
PMT	polyethylene glycol (PEG)-mediated transformation
CRISPR	Clustered Regularly Interspaced Short Palindromic Repeats
sgRNA	single-guide RNA
RNP	ribonucleoprotein
PB	particle bombardment
